# Identification of novel, cryptic *Clostridioides* species isolates from environmental samples collected from diverse geographical locations

**DOI:** 10.1099/mgen.0.000742

**Published:** 2022-02-15

**Authors:** Charles Hall Davis Williamson, Nathan E. Stone, Amalee E. Nunnally, Chandler C. Roe, Adam J. Vazquez, Samantha A. Lucero, Heidie Hornstra, David M. Wagner, Paul Keim, Maja Rupnik, Sandra Janezic, Jason William Sahl

**Affiliations:** ^1^​ Pathogen and Microbiome Institute, Northern Arizona University, PO Box 4073, Flagstaff, AZ 86011, USA; ^2^​ National Laboratory for Health, Environment and Food, Prvomajska Ulica 1, 2000 Maribor, Slovenia; ^3^​ Faculty of Medicine, University of Maribor, Taborska 8, 2000 Maribor, Slovenia

**Keywords:** *Clostridioides difficile*, cryptic species, genomics, toxin

## Abstract

*
Clostridioides difficile* is a pathogen often associated with hospital-acquired infection or antimicrobial-induced disease; however, increasing evidence indicates infections can result from community or environmental sources. Most genomic sequencing of *C. difficile* has focused on clinical strains, although evidence is growing that *C. difficile* spores are widespread in soil and water in the environment. In this study, we sequenced 38 genomes collected from soil and water isolates in Flagstaff (AZ, USA) and Slovenia in an effort targeted towards environmental surveillance of *C. difficile *. At the average nucleotide identity (ANI) level, the genomes were divergent to *C. difficile* at a threshold consistent with different species. A phylogenetic analysis of these divergent genomes together with *Clostridioides* genomes available in public repositories confirmed the presence of three previously described, cryptic *Clostridioide* s species and added two additional clades. One of the cryptic species (C-III) was almost entirely composed of Arizona and Slovenia genomes, and contained distinct sub-groups from each region (evidenced by SNP and gene-content differences). A comparative genomics analysis identified multiple unique coding sequences per clade, which can serve as markers for subsequent environmental surveys of these cryptic species. Homologues to the *C. difficile* toxin genes, *tcdA* and *tcdB*, were found in cryptic species genomes, although they were not part of the typical pathogenicity locus observed in *C. difficile *, and *in silico* PCR suggested that some would not amplify with widely used PCR diagnostic tests. We also identified gene homologues in the binary toxin cluster, including some present on phage and, for what is believed to be the first time, on a plasmid. All isolates were obtained from environmental samples, so the function and disease potential of these toxin homologues is currently unknown. Enzymatic profiles of a subset of cryptic isolates (*n*=5) demonstrated differences, suggesting that these isolates contain substantial metabolic diversity. Antimicrobial resistance (AMR) was observed across a subset of isolates (*n*=4), suggesting that AMR mechanisms are intrinsic to the genus, perhaps originating from a shared environmental origin. This study greatly expands our understanding of the genomic diversity of *Clostridioides *. These results have implications for *C. difficile* One Health research, for more sensitive *C. difficile* diagnostics, as well as for understanding the evolutionary history of *C. difficile* and the development of pathogenesis.

## Data Summary

Genomic sequence data generated as part of this study have been deposited at the National Center for Biotechnology Information (NCBI) under BioProject accession numbers PRJNA438482 and PRJNA662350, with specific accession numbers as shown in Table S1. Public genomic data were downloaded from NCBI databases (Table S2).

**Impact Statement**

*
Clostridioides difficile* is a pathogen often associated with severe diarrhoea that can be difficult to treat and can cause potentially fatal infections in humans. Understanding the diversity and ecology of *C. difficile* and closely related bacteria is important from a public-health perspective, as this knowledge could aid in identifying sources of human infections and could improve diagnostics for detecting *C. difficile *. In this study, we used comparative genomic analyses to characterize bacterial isolates collected from environmental samples from distant geographical locations. The isolates are closely related to *C. difficile* but can be considered multiple, different species, and some isolates contain homologues of *C. difficile* toxin genes. This study contributes to our understanding of the diversity of *Clostridioides* and can provide a framework for studying pathogenic members of the genus.

## Introduction

*Clostridioides difficile* is an anaerobic, spore-forming pathogen that can cause potentially fatal infections in humans. Although *C. difficile* has been associated with nosocomial infections and the human gut, the organism has been isolated from a variety of sample types and environments including non-human animals, water (including wastewater treatment facilities), household environments, and soil from urban or rural settings [1–3]. These strains are genetically similar to those isolated from humans in clinical settings, suggesting that the same strains can inhabit multiple reservoirs and that the environment is a source of human infections.

The use of whole-genome sequencing is changing our view of *C. difficile* composition, evolution and differentiation. In 2016, divergent types of *C. difficile* were observed in environmental samples based on multilocus sequence typing (MLST) analysis and ribotyping [4]. In 2014, the cryptic clade C-I was identified, although at the time only non-toxigenic strains were described [5]. In 2018, a core-genome MLST system identified the presence of two additional cryptic lineages (C-II and C-III) [6]. A recent study used average nucleotide identity (ANI) to demonstrate that each of these lineages is distinct from each other and *C. difficile *, representing unique genomospecies [7]. The function, geographical distribution and complete genomic complement of genomes from these lineages are understudied.

Divergent lineages were also reported to contain unique toxin-encoding regions. Three toxins are produced by *C. difficile* toxigenic strains in various combinations. Toxins A and B, encoded by *tcdA* and *tcdB*, respectively [8], are accepted as the main virulence factors [9], whereas the role of the binary toxin CDT has not been fully determined [10, 11]. Toxins A and B are encoded by genes in the PaLoc region, which in nontoxigenic strains is replaced by a section of DNA 115 bp in length; in divergent strains, large insertions have been observed instead of the PaLoc [4, 5, 12]. Although *tcdB+* strains that lack *tcdA* are fully virulent in animal models [13], strains that lack *tcdB* can cause disease [13] but appear to be attenuated [14]. There have been numerous examples of *tcdB*+/*tcdA− C. difficile* isolates [15]. Although *tcdB*−/*tcdA+* isolates of *C. difficile* have not been reported, this profile has been found in related cryptic isolates [16].

Identification of *C. difficile* in the clinic typically includes the detection of either the *tcdB* or the *tcdA* genes [17, 18], the 16S rRNA gene [19] and/or the *tpi* gene (triose phosphate isomerase) [20] through PCR or the detection of toxins A and B and/or glutamate dehydrogenase (GDH) with enzyme immunoassays [21, 22]. *C. difficile* have been isolated from the environment through culture-based methods using selective media [23], as the pathogen is expected to be a minor component of the microbial community. Isolates are characterized with diverse methods including colony morphology [24], MALDI-TOF [4] and molecular methods such as PFGE [25] or ribotyping [4, 26]. Following enrichment and/or isolation, many workflows focus on the detection of *tcdA* [27] and *tcdB* [28] to identify *C. difficile *. However, *C. difficile* sampling that focuses on detection of toxin genes could miss the diversity of *Clostridioides* that don’t possess these genes or have nucleotide differences that could evade current molecular assays. Currently, genomic characterization of environmental *C. difficile* isolates is less common than for clinical isolates [7]. For clinical and environmental detection of *C. difficile *, understanding the genomic diversity of the pathogen, as well as genetic near neighbours, is crucial for ensuring appropriately sensitive and specific assays. To better understand the genomic landscape of divergent *Clostridioides* lineages, we describe a comparative genomics analysis of cryptic *Clostridioides* isolates collected from environmental samples in two diverse geographical locations: Flagstaff, (AZ, USA) and Slovenia (Europe).

## Methods

### Sample collection, bacterial cultivation, DNA extraction and sample screening

The sampling and isolation of strains from Slovenia has been previously described [4, 29]. To survey *C. difficile* throughout Flagstaff, AZ, USA, soil (*n*=210) and water (*n*=50) samples were collected at various locations (Fig. S1, Table S1, available with the online version of this article). Two rounds of sampling were conducted between 2017 and 2019. For the first round of sampling, 50 soil and 50 water samples were collected in 2017. Soil samples were collected from the top 2 inches (5 cm) of soil using a disposable plastic spoon and stored in a quart-sized plastic bag at 4 °C until processing. For water samples, a maximum of 150 ml water was collected from each location. The water samples were filtered using a Combisart three branch manifold (Sartorius) with 250 ml sterile funnels containing Microsart filters (cellulose nitrate, 47 mm diameter, 0.2 µM pore size) and a Microsart EJet pump. Sterile Minisart syringe filters (25 mm, 0.2 µm PTFE) were attached to each branch on the apparatus for sterile venting. The filter was stored at 4 °C until processing. Two grams of soil or one-half of a cellulose nitrate membrane filter was incubated in Cdiff banana broth (Hardy Diagnostics) at 36 °C for 72 h. A 200 µl aliquot of enrichment culture was plated onto taurocholate-cefoxitin-cycloserine-fructose agar (TCCFA) and incubated for 24 h at 36 °C in a vinyl type C anaerobic chamber (Coy Laboratory Products). A 20 % (v/v) glycerol stock was created from this population for future work, and DNA was extracted from the population using a Qiagen DNeasy blood and tissue kit, and screened with Taqman PCR assays designed to detect *C. difficile* and the presence of the *tcdB* gene [30]. The *C. difficile* Taqman PCR assay includes primers targeting a unique *C. difficile* marker (CD630_24840) used previously for detection of *C. difficile* in dog faecal samples [30]. Samples with positive Taqman PCR assay results for the *C. difficile* marker were streaked for isolation on brain heart infusion agar supplemented with 0.03 % l-cysteine (BHIS) and incubated anaerobically for 24 h at 36 °C. Lawns were created for each isolate and DNA was extracted using a Qiagen DNeasy blood and tissue kit for whole-genome sequencing. For the second round of sampling in Flagstaff (160 soil samples collected in 2018 and 2019), approximately 3 g soil collected from the top 1 inch (2.5 cm) of sampling sites was added to Cdiff banana broth and incubated at 37 °C for 72 h. From these enrichments, glycerol stocks were created and DNA was extracted with a MoBio Powersoil DNA extraction kit. Samples with positive results for the *C. difficile* Taqman PCR assay were streaked for isolation on TCCFA; colonies were picked and streaked onto BHIS, and DNA was subsequently extracted with a Qiagen DNeasy blood and tissue kit for whole-genome sequencing.

### Sequencing, genome assembly and MLST

All DNA was sequenced on the Illumina MiSeq platform; for a subset of genomes, sequencing was also performed on the Nanopore MinION platform. DNA extracted from Flagstaff isolates was prepared for multiplexed, paired-end sequencing with a 500 bp insert using standard PCR library amplification (KAPA Biosystems). DNA extracted from Slovenian isolates was prepared using the Nextera XT DNA library preparation kit (Illumina). Genomes were sequenced on the Illumina MiSeq platform. Illumina data was adapter trimmed with BBDuk v38.86 (https://sourceforge.net/projects/bbmap/), assembled with Spades v3.10.0 or v3.14.0 [31], polished with Pilon v1.22 [32] and contigs >500 nt were annotated with Prokka v1.14.6 [33]. For MinION sequencing, samples were streaked for isolation from glycerol stocks using BHIS and incubated anaerobically at 37 °C for 24 h; high molecular mass DNA was extracted using the Quick-DNA HMW MagBead kit (Zymo). DNA was assessed for quality using a standard genomic 50 kb Fragment Analyzer kit (Agilent) to ensure mean DNA fragments >60 000 kb. Libraries were prepared using a SQK-LSK109 1D ligation gDNA kit with the native barcoding gDNA kit (Oxford Nanopore). Libraries were loaded onto a R9/R9.4 flow cell and MinION sequencing was performed for 60 h using MinKNOW software. Guppy v3.22 was used for basecalling using the 9.4.1_450bps_hac workflow, and reads were trimmed with Porechop v0.2.4 (https://github.com/rrwick/Porechop). Unicycler v0.4.8 [34] was used to generate hybrid assemblies where Illumina and MinION data were available. MLST profiles of sequenced genomes were determined by stringMLST v0.6.3 (-k 45) [35].

### Publicly available genomic data

Genome assemblies matching the search term ‘Clostridioides’ were downloaded from GenBank on 20 October 2020 (*n*=2520). Two of these genome assemblies were discarded due to anomalous G+C content (the excluded genomes had G+C content >44 mol%, the G+C content of included genomes ranged from 28.04–32.78 mol%). Paired-end Illumina reads for isolates identified as belonging to cryptic *Clostridioides* lineages by Knight *et al*. [7] were downloaded from the National Center for Biotechnology Information (NCBI) Sequence Read Archive (SRA). To identify additional genomes belonging to cryptic *Clostridioides* lineages, the SRA was also searched for ‘Clostridioides difficile’ on 20 October 2020 and reads released after 1 January 2020 were downloaded (this set of reads includes data released after the 2020 Knight *et al*. publication [7]). MLST profiles were assigned to paired-end Illumina data with stringMLST v0.6.3 (-k 45) [35]. Reads with MLST profiles known to be associated with *C. difficile* clades 1–5 were discarded. The remaining reads were assembled as described above and assemblies with anomalous genome size or G+C content were discarded (the assembly size and G+C content of included genomes are listed in Table S2). Genome assemblies were then compared with mash v2.1 [36] and only genomes associated with cryptic *Clostridioides* lineages closely related to *C. difficile* were retained. The final data set included 2518 GenBank assemblies and 28 genomes downloaded from the SRA (Table S2).

### ANI

After an initial screening of genomes with mash, a set of 101 reference genomes was chosen from the entire data set (all genomes in Table S1 and 63 genomes in Table S2); this smaller set of genomes included all genomes associated with cryptic *Clostridioides* lineages and representative genomes from each *C. difficile* clade (1–5) (Tables S1 and S2). These genomes were compared with pyani (v0.2.10 – https://github.com/widdowquinn/pyani) [37], using the MUMmer v3.23 [38] alignment option. An ANI (ANIm value) of 95 % was used to delineate species/lineage boundaries, which is consistent with published criteria [39].

### Core-genome SNP phylogeny

A core-genome SNP analysis was performed for the set of 101 reference genomes. All genomes were aligned against the completed *C. difficile* genome CD630 (GenBank accession no. NC_009089) using NUCmer v3.1 [40] and SNPs were called with nasp v1.2 [41]. Any SNP that fell within a duplicated region in the CD630 genome, based on a self-alignment with NUCmer, was filtered from downstream analyses. A phylogeny was inferred from a concatenation of SNPs with iq-tree v2.0.3 [42], using the TN+F+ASC+R3 substitution model [43].

A core-genome SNP analysis was also performed on C-III genomes within nasp by aligning Illumina sequencing reads to the completed assembly for ES-W-0016-02 (GenBank accession numbers CP061361 and CP061362) with bwa-mem v0.7.17 [44], and calling SNPs with the UnifiedGenotyper method in gatk v3.4-46 [45, 46]. SNPs were filtered from downstream analyses if the depth of coverage was less than 5x, the proportion of base calls was less than 0.9, the SNP was identified in a duplicated region of the reference genome, or if the SNP was in close proximity to another SNP call (within 5 positions), which may indicate a low-quality SNP call [47]. A phylogeny was generated with iq-tree as described above (substitution model TVM+F+R3, SNPs queried from *C. difficile* CD630 were used to root the phylogeny).

### Comparative genomics and pan-genomics

To determine the sizes of the pan and core genomes of the novel clades, ‘.gff’ files produced by Prokka were processed by Panaroo v1.2.3 [48] using default settings. Coding region sequences (CDSs) were considered to be part of the core genome if they were conserved in >99 % of genomes surveyed. CDSs unique to each clade were identified with the Large-scale blast Score Ratio (ls-bsr) pipeline v1.2.2 [49]. CDSs for a representative genome in each clade were screened against all genome assemblies (*n*=2584) using the blastn alignment option. A CDS was considered a lineage-specific marker if the CDS had a blast score ratio (BSR) [50] value ≥0.8 for all in-group genome assemblies and a BSR value <0.4 for all out-group genome assemblies. Unique regions were translated with Biopython and the clusters of orthologous groups (COG) annotation was determined by eggNOG-Mapper v2.0.1–14 [51].

In addition, the protein sequences for genes associated with toxins in *C. difficile* were screened against the reference genome set (*n*=101) with tblastn v2.10.0+ [52] in conjunction with ls-bsr. The resulting matrix was converted into a binary presence (‘1’) or absence (‘0’) matrix based on a BSR threshold of 0.5; this is equivalent to 50 % identity over 100 % of the protein length. The resulting heatmap was visualized with the Interactive Tree of Life [53]. The gene order of the PaLoc and the CdtLoc in selected genomes was visualized by genoPlotR [54].

### Plasmid and phage screen

Previously published sequences from *C. difficile* plasmids [55] and phage [56] were downloaded from GenBank; additional extrachromosomal regions were downloaded based on the study by Hornung *et al*. [57]. Circular contigs assembled in this study that were not associated with the chromosome were added to these other sequences and a dendrogram was created with MashPy, a pipeline that clusters pairwise mash distances (https://gist.github.com/jasonsahl/24c7cb0fb78b4769521752193a43b219).

### Enzymatic profiles of isolates from new clades

Enzymatic profiles for five isolates representing newly identified clades were evaluated using the API 20A system (bioMérieux). Five anaerobic isolates were grown overnight on BHIS at 37 °C within an anaerobic chamber. After 24 h, isolates were suspended in Mueller Hinton broth (Millipore Sigma) to a 1 McFarland turbidity standard. Using this suspension, bacterial lawns were created on Brucella blood agar with haemin and vitamin K media (Hardy Diagnostics). Isolates were grown on blood media overnight at 37 °C for 24 h under anaerobic conditions. Samples were suspended in inoculation medium API 20A (Anaerobe) from API system (bioMérieux) to a 3 McFarland turbidity standard and then added into microtubes according to the manufacturer’s instructions. The API 20A strip consisted of 20 microtubes with dehydrated substrates. All test strips were incubated at 37 °C with humidity overnight and tests were scored after 24 h. These reactions were scored according to the manufacturers 'Reading Table'. The following tests were determined: gelatine (GEL), urea (URE), aesculin hydrolysis (ESC), indole synthesis (IND) and the fermentation ability of 16 carbohydrates – d-glucose (GLU), d-mannitol (MAN), d-lactose (LAC), d-sucrose (SAC), d-maltose (MAL), salicin (SAL), d-xylose (XYL), l-arabinose (ARA), glycerol (GLY), d-cellobiose (CEL), d-mannose (MNE), d-melezitose (MLZ), d-raffinose (RAF), d-sorbitol (SOR), l-rhamnose (RHA) and d-trehalose (TRE). Additionally, a catalase reaction in which the addition of hydrogen peroxide (3%) was added to culture and the presence (positive result) or absence (negative result) of bubbles was recorded.

### Antimicrobial-resistance (AMR) profiles of select isolates

Four cryptic *Clostridioides* isolates representing two cryptic lineages were screened for AMR against the following drugs known to induce *C. difficile* infection (CDI): ceftriaxone, ceftazidime, clindamycin, ciprofloxacin, imipenem, cefotaxime, cefepime, erythromycin, moxifloxacin and levofloxacin. Minimum inhibitory concentrations (MICs) were determined across these ten antimicrobials using ETESTs (bioMérieux) as per the Clinical and Laboratory Standards Institute (CLSI) 2020 guidelines, where available; for ciprofloxacin, no breakpoint was listed and the breakpoint for moxifloxacin was used, as has been done previously [58]. Samples were streaked for isolation from glycerol stocks using BHIS and incubated anaerobically at 37 °C for 24 h. An initial inoculum was prepared by making a Mueller Hinton broth suspension of isolated colonies from the overnight growth on BHIS plates. The bacterial suspension was adjusted to achieve turbidity equivalent to a 1.0 McFarland turbidity standard (Remel). Bacterial lawns were created from this suspension using a sterile swab and Brucella blood agar with haemin and vitamin K (Hardy Diagnostics) according to standard protocols. ETESTs were applied on plates following a 10 min dry time and plates were incubated at 37 °C for 48 h, after which MICs were scored according to the manufacturer’s instructions. All MIC testing was performed in biological duplicate.

### *In silico* predicted AMR profiling

The set of 101 reference genomes were screened with the Comprehensive Antimicrobial Resistance Database (CARD) [59] genes with blastn and ls-bsr. Any CARD region with a BSR ≥0.75 was considered to be conserved. Protein sequences of interest (GyrA and GyrB) were identified with tblastn v2.9.0+ [52], aligned with muscle v3.8.31 [60] and examined for mutations associated with AMR listed in the CARD database [59].

### 16S rRNA gene and *rpoB* gene analyses

16S rRNA gene sequences were extracted from Prokka annotation files for the reference set genomes (*n*=101). For genome assemblies with multiple 16S copies, a single sequence was selected for each genome (the longest clostridial 16S gene sequence for each genome). No 16S rRNA gene sequence was retrieved for two genomes in the reference set. Sequences were aligned with muscle v3.8.31 [60] and a maximum-likelihood phylogeny was inferred with iq-tree v1.6.12 [61] using the selected best fit substitution model (HKY+F+R2) [43]. The V4 region of the 16S rRNA gene commonly targeted in microbiome research (515F, 806R) [62] was extracted from sequences and analysed as described above (amplicon sequences were predicted for 89 genomes and included in this analysis, substitution model JC). *rpoB* gene sequences were also extracted from Prokka annotation files and aligned with muscle, followed by generation of a maximum-likelihood phylogeny with iq-tree (substitution model TIM2+F+I+G4). To identify fragments of the *rpoB* gene that would be a good target for clade differentiation, the *rpoB* gene was split into 300 bp fragments and processed with Phylomark v1.6 [63] in order to find the fragment that best separated the clades; a phylogeny was inferred from the resulting alignment with FastTree2 v2.1.10 [64].

### *In silico* PCR

Primers and probes associated with different targets (Table S3) were screened against the reference genome set (*n*=101) with usearch (v11.0.667_i86linux32) [60] using the search_pcr and search_oligodb commands with default settings. The primers screened include those that target the signature previously determined to be unique to *C. difficile* [30], two sets of primers targeting four toxins [65, 66] and a primer set that we developed for more inclusive *tcdB* detection (Table S3).

## Results

### Sample collection, isolation and genome sequencing

A total of 260 samples were collected from soil and water around Flagstaff (AZ, USA) (Fig. S1, Table S1), in an effort targeted towards *C. difficile* surveillance. Positive results for a Taqman PCR assay targeting *C. difficile* [30] were observed for approximately 50 % of the 260 environmental samples, and isolates from these samples were whole-genome sequenced. Twenty-one of these sequenced isolates either had a multilocus sequence type (ST) associated with cryptic *Clostridioides* species or had a novel ST and were included in this study. The 21 isolates originated from 19 samples, as 2 samples included isolates with two multilocus STs associated with cryptic clades. Additional studies focusing on *C. difficile* isolated from the Flagstaff sampling effort are in progress. Slovenian isolates with STs associated with cryptic clades were collected from soil (*n*=14), puddle water (*n*=1) [4] and dandelion (*n*=2) [29], resulting in 17 genomes.

### Sequenced genome clade designation

**Fig. 1.**
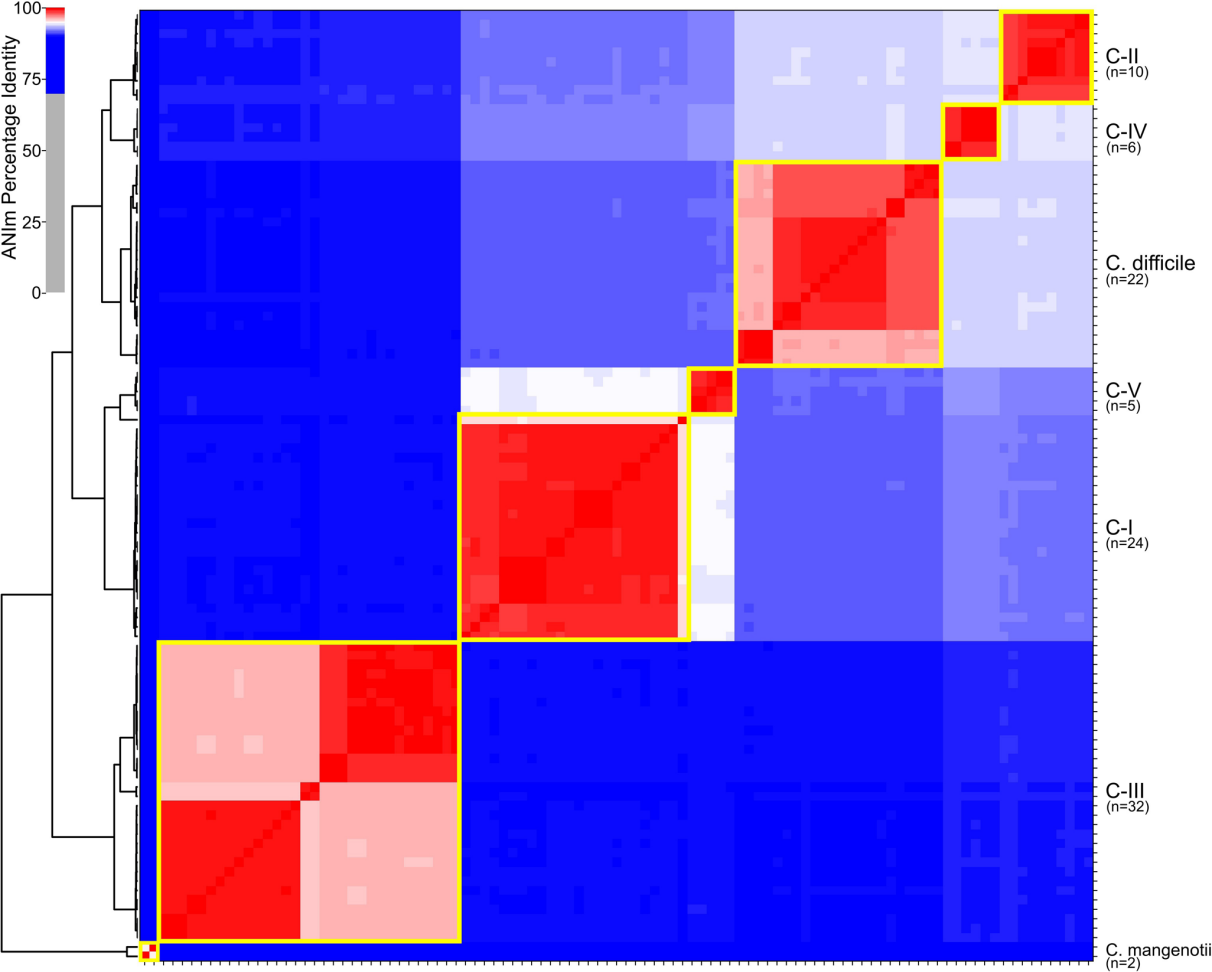
Heat map and clustering of genomes based upon pairwise ANI values (ANIm value computed with pyani [37]). Yellow boxes indicate species/lineage boundaries of 95 % ANI.

Following sequencing and genome assembly, an initial screen was performed using mash to verify assignment of genomes into cryptic clades. After this initial screen, a set of 101 genomes that included all cryptic species genomes and representative genomes from *C. difficile* were analysed with pyani (Table S4). The results demonstrate the presence of five defined clades (C-I–C-V, within each clade ANI values are >95 %) that are distinct from *C. difficile* (Fig. 1). The pairwise pyani distances (Table S4) between clades demonstrates that each clade represents a separate species based on published thresholds [39], confirming recently published results [7]. Isolate genomes from Flagstaff fall within two of these novel clades, including one that has not been described previously (clade C-IV). One of these novel clades (C-III) primarily includes genomes from environmental isolates collected in rural eastern Slovenia [4] and Flagstaff (AZ, USA). Genomes from Slovenia isolates fall into clades C-I, C-II and C-III.

### Core-genome phylogeny and toxin screen

**Fig. 2.**
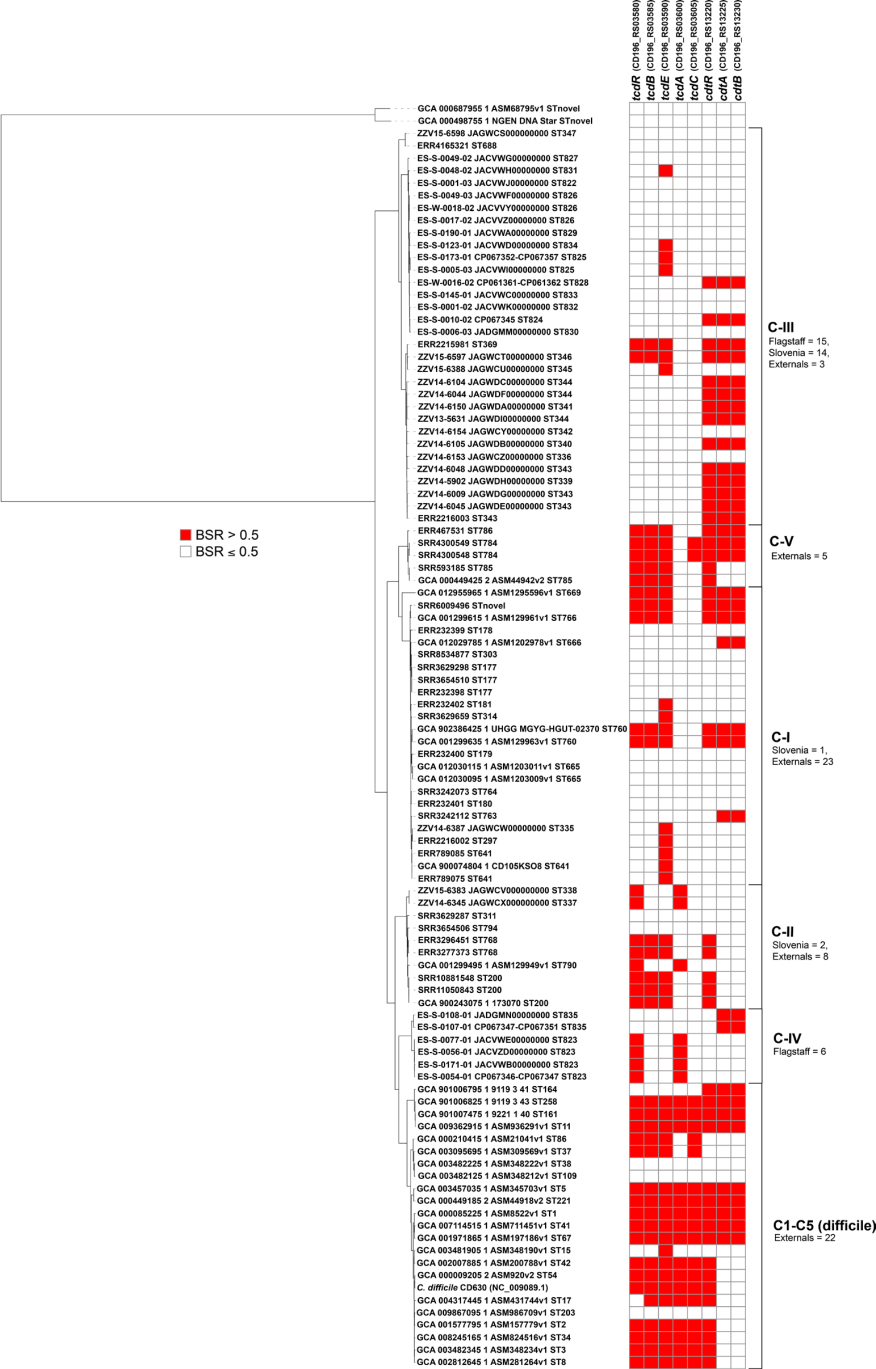
A core-genome, maximum-likelihood SNP phylogeny of 101 reference genomes rooted with *C. mangenotii* (top of phylogeny) demonstrating a similar topology to the clustering of genomes with ANI values. The presence or absence of *C. difficile* toxins (*tcdR*, *tcdB*, *tcdE*, *tcdA*, *tcdC*, *cdtR*, *cdtA*, *cdtB*) is displayed in the heat map to the right of the phylogeny. Toxin presence or absence was determined with a BSR cut-off value of 0.5 (see Methods). The numbers of genomes from Flagstaff (this study), Slovenia (this study) or downloaded from the NCBI (labelled as external) in each species/lineage are listed next to the heat map.

A core-genome phylogeny rooted with *Clostridium. mangenotii* (Fig. 2) demonstrated a similar topology to the pyani distance cluster. *C. difficile* toxin sequences were screened against the reference genome set and the presence/absence of each toxin (based on BSR value cut-off of 0.5, see Methods) was correlated with each genome (Fig. 2). The results demonstrate that there are homologues to *tcdB* in clades C-I, C-II, C-III and C-V. There were also homologues to *tcdA* in clades C-II and C-IV. There were homologues to binary toxins (*cdtA*, *cdtB*) in all cryptic clades except for C-II.

**Fig. 3.**
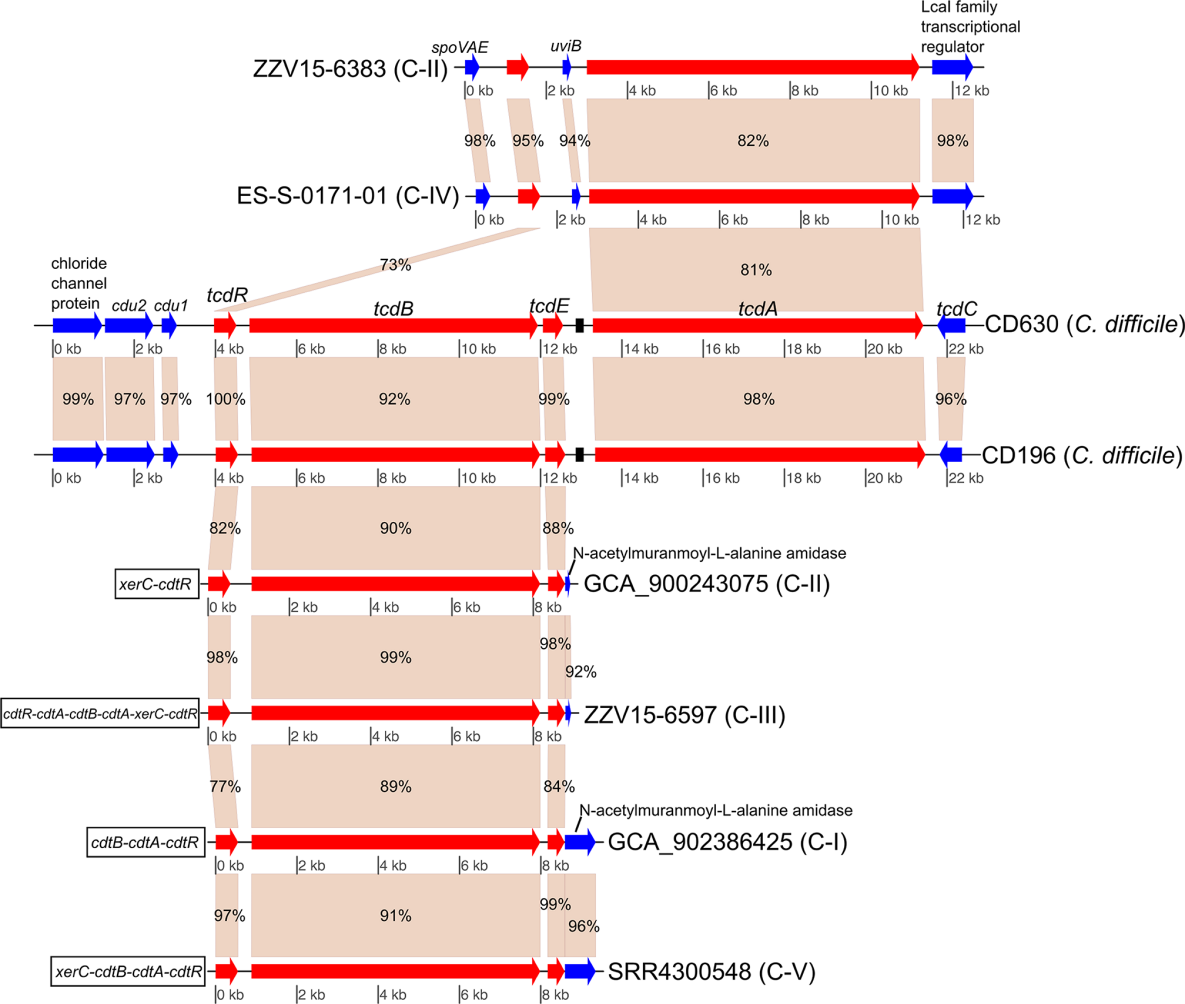
Gene map of *tcdB* and *tcdA* regions in genomes from various species/lineages containing *C. difficile* toxin homologues. Toxin gene homologues (*tcdR*, *tcdB*, *tcdE*, *tcdA*) are displayed in red, and flanking genes are displayed in blue. *tcdB* homologues were observed in genomes in groups C-I, C-II, C-III and C-V, and *tcdA* homologues were observed in genomes in groups C-II and C-IV. No cryptic lineage genomes contained both *tcdB* and *tcdA* homologues. The figure was generated with genoPlotR [54]. Numbers on genes indicate pairwise blast identities.

For cryptic clade genome assemblies in which homologues to *tcdB* were observed (groups C-I, C-II, C-III and C-V), no homologue to *tcdA* was identified. However, homologous flanking genes were observed in many of the cryptic species genome assemblies containing *tcdB* homologues (Fig. 3), suggesting common integration sites not associated with the traditional *C. difficile* PaLoc. Genes associated with the binary toxin locus were present adjacent to the *tcdB* gene cluster, which has been described elsewhere [7, 67]. In cryptic clade genomes with homologues to *tcdA* (C-II and C-IV), the *tcdA* genes were also not part of the traditional PaLoc gene cluster. These novel *tcdA* clusters contained similar proteins in the same gene order despite the isolates originating from regions separated by large geographical distances. One of these isolates was collected from the USA and the other isolate was collected from Slovenia; another C-II genome (accession no. GCF_001299495.1) from isolate RA09-70, collected in Paris, France, had the identical gene structure to these two genomes. In these clusters, *tcdR* was upstream of the *tcdA* homologue, although another coding region (*uviB*) was found directly adjacent to the *tcdA* homologue.

**Fig. 4.**
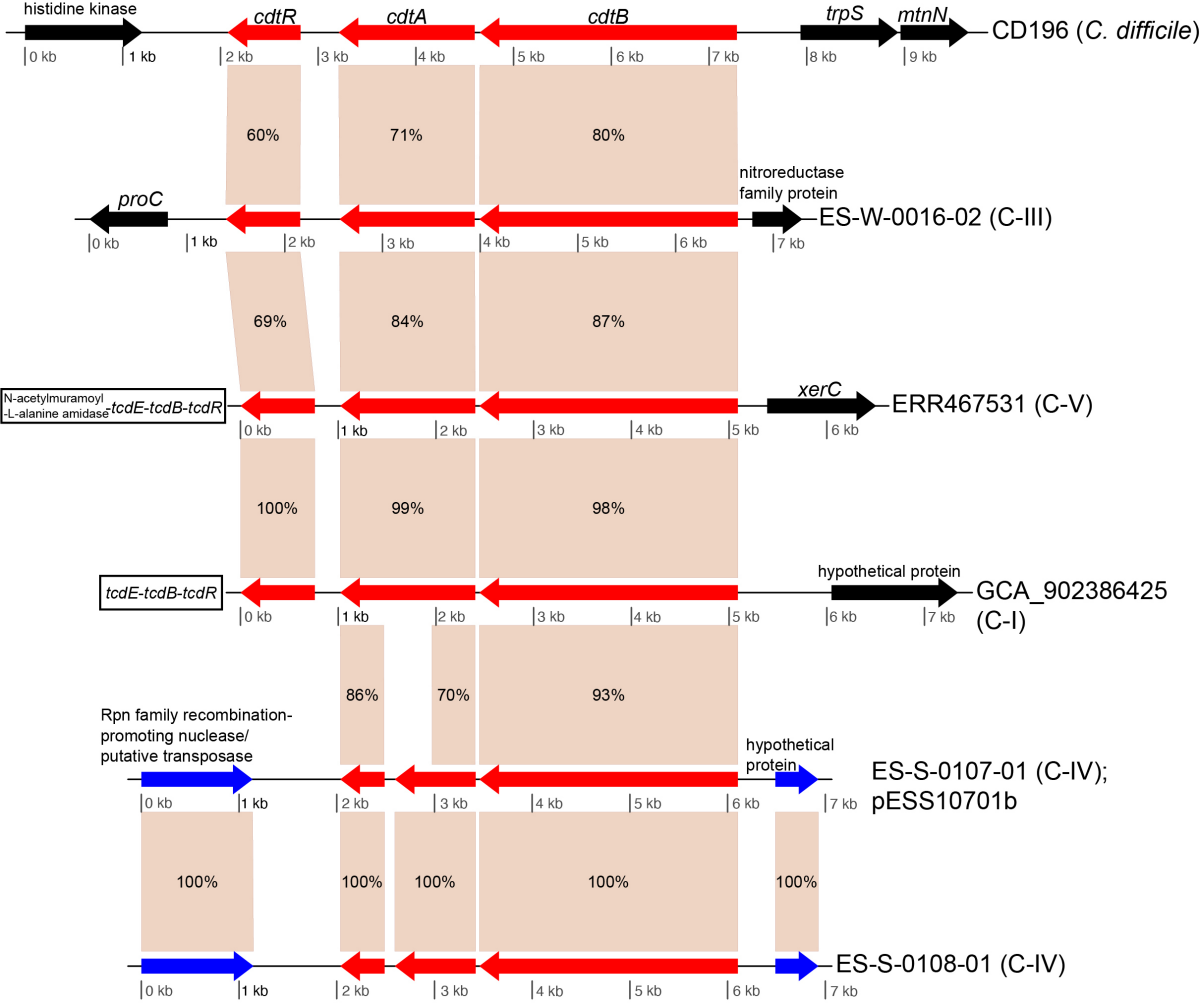
Gene map of binary toxin gene regions in genomes from various species. Binary toxin homologues are displayed in red. Binary toxin gene homologues were observed in isolates from clades C-I, C-III, C-IV and C-V. The figure was generated with genoPlotR [54]. Numbers on genes indicate pairwise blast identities. Blue arrows indicate homologous genes outside of the toxin cluster while black boxes indicate genes with no homolog in other gene clusters.

For the binary toxin cluster, CdtLoc, homologous genes were observed across isolates from clades C-I, C-III, C-IV and C-V (Fig. 4). Two of the genomes in clade C-IV contained homologues for *cdtA* and *cdtB*, but were missing *cdtR*. An investigation of the gene structure of these genomes demonstrated that the *cdtA* homologue was split between two predicted coding regions. The entire locus was identified on a plasmid in isolate ES-S-0107-01, suggesting mobile transport and explaining the lack of the locus in other genomes from the clade. ES-S-108-01 was isolated from a sample taken on the same day as ES-S-0107-01, but from a different location, and demonstrated the same gene structure as ES-S-0107-01. Although we only have a draft genome assembly for isolate ES-S-0108-01, the similarity in gene content to ES-S-107-01, as well as the coverage of the pESS10701b plasmid (Fig. S2), suggests that the binary toxin homologues also are found on an extrachromosomal element in this isolate.

### Pan-genomics of novel clades

Panaroo was used to calculate the pan and core genome for each novel clade (Table 1). Unique sequences were identified for most lineages with ls-bsr (Tables 1 and S5), which is consistent with previous work [7]. For each clade, the pan-genome size is much larger than the core-genome size, suggesting that the pan-genomes are being driven by the acquisition of exogenous genes in the environment, which is seen in some other pathogenic bacteria such as *Burkholderia pseudomallei* [68].

**Table 1.** Pan-genomic information for cryptic species genomes

Clade	No. of genomes	Core genome	Pan-genome	Unique regions
C-I	24	3039	6369	5
C-II	10	2891	5148	0
C-III	32	3014	8135	53
C-IV	6	3073	4390	40
C-V	5	3626	5133	27

**Fig. 5.**
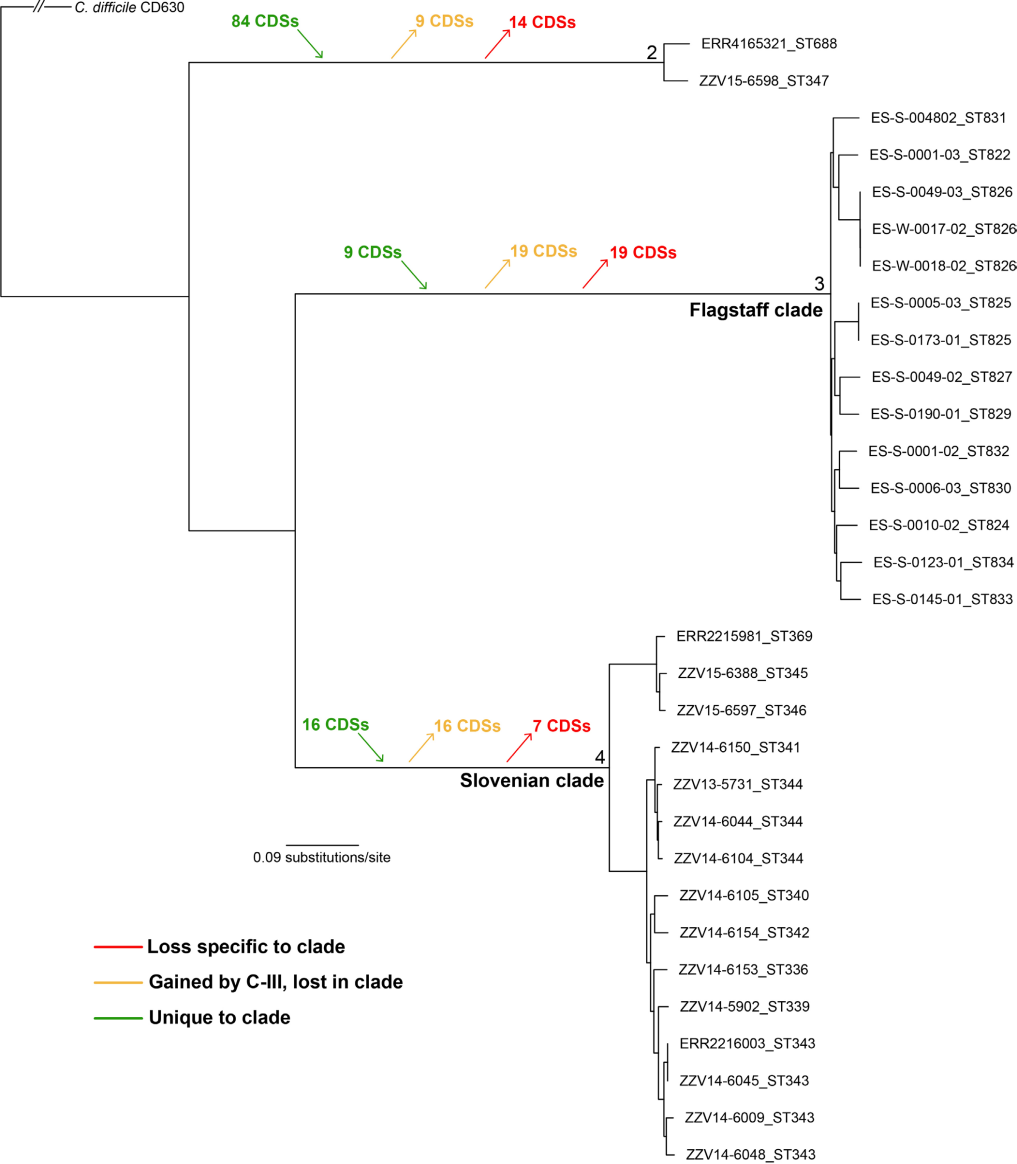
A core-genome, maximum-likelihood SNP phylogeny of C-III genomes displaying coding regions conserved or lost among lineages. Unique coding regions are present in all three major lineages of C-III, based on an analysis with ls-bsr (see Methods). The phylogeny was rooted by querying SNPs in *C. difficile* CD630.

A comparative pan-genomics analysis was also performed between Flagstaff and Slovenian genomes in clade C-III, which fell into two distinct clades (differentiated by thousands of SNPs) in a core-genome SNP phylogeny (Fig. 5). The results demonstrate the presence of 9 unique coding regions to the Flagstaff clade, 16 unique coding regions to the large Slovenian clade, and 84 regions unique to a small clade including a genome from Slovenia and a genome from an isolate collected from septic arthritis (ERR4165321) (Fig. 5, Table S6). Other regions were identified that were highly conserved in *Clostridioides* genomes, but lost in all C-III genomes, as well as regions that were only conserved in C-III genomes, but were then lost in sub-clades (Fig. 5, Table S6).

### Completed representative genomes from novel clades

Finished genomes were generated for five isolates from groups C-III and C-IV. For three of the isolates, plasmids were identified (Table 2). For four of the strains, phage sequences were identified from both *Myoviridae* and *Siphoviridae*, based on a cluster dendrogram with previously published and annotated phage (Fig. S3). All assignments of phage or plasmid were based on sequence similarity to these previously characterized elements. Reads from all genomes sequenced in this study were mapped against all extrachromosomal elements and the breadth of coverage was calculated (Fig. S2). The phiSemix9P1 phage (KX905163.1) was present in multiple clade C-III genomes from Slovenia; this phage contains genes from the binary toxin cluster, including *cdtR*, *cdtA* and *cdtB*.

**Table 2.** Finished genome assembly information

Genome	Accession no.	Element type	Element length (bp)
ES-W-0016-02	CP061361	Chromosome	4 189 111
	CP061362	Plasmid pESW1602	305 867
	CP069348	Phage pCD1602_4	132 519
ES-S-0010-02	CP067345	Chromosome	4 259 110
ES-S-0173-01	CP067353	Chromosome	4 100 889
	CP067354	Plasmid pESS17301a	136 427
	CP067355	Plasmid pESS17301c	47 667
	CP067356	Plasmid pESS17301d	11 693
	CP067357	Plasmid pESS17301e	4119
	CP067352	Phage ES-S-0173-01	113 019
ES-S-0054-01	CP067346	Chromosome	3 925 358
	CP069347	Phage pCD5401_3	34 243
ES-S-0107-01	CP067348	Chromosome	3 896 990
	CP067349	Plasmid pESS10701b	105 438
	CP067350	Plasmid pESS10701c	64 238
	CP067351	Plasmid pESS10701d	18 539
	CP067347	Phage ES-S-0107-01	132 924

### Biochemical characterization

Five Flagstaff isolates were processed with the API 20A kit (bioMérieux) in order to identify enzymatic differences between isolates and to compare the isolates to published results for *C. difficile *. According to the manufacturer’s documentation, at least some *C. difficile* isolates have produced positive results for 12 of the included reactions; many of these reactions are not commonly reported as positive for *C. difficile* (see the manufacturer’s documentation). Positive results were observed for at least one of the tested isolates for all reactions except MLZ (Table 3). Only one isolate, ES-W-0016-02, produced positive results for reactions not reported for *C. difficile* in the manufacturer’s documentation; for this isolate, positive reactions were observed for LAC, SAC, MAL, ARA and catalase production (CAT). The results indicate that differentiating these cryptic lineages based solely on this assay may be difficult; however, the results also demonstrate that there is variation within and between groups (Table 3), suggesting that these isolates are capable of different metabolic roles.

**Table 3.** Biochemical characterization of Flagstaff isolates Grey highlighting indicates reactions that have been observed to be positive in at least some *C. difficile* isolates according to the manufacturer.

Sample	Clade	IND	URE	GLU	MAN	LAC	SAC	MAL	SAL	XYL	ARA	GEL	ESC	GLY	CEL	MNE	MLZ	RAF	SOR	RHA	TRE	CAT
H_2_O	na	−	−	−	−	−	−	−	−	−	−	−	−	−	−	−	−	−	−	−	−	−
ES-S-0010-02	C-III	−	−	+	+	−	−	−	−	−	−	+	+	−	−	+	−	−	−	−	−	−
ES-W-0016-02	C-III	−	−	+	+	+	+	+	+	+	+	+	+	−	+	+	−	−	+	+	+	+
ES-S-0173-01	C-III	−	−	+	+	−	−	−	+	−	−	+	+	−	−	+	−	−	−	−	−	−
ES-S-0054-01	C-IV	−	−	+	+	−	−	−	−	−	−	−	+	−	−	+	−	−	+	−	+	−
ES-S-0107-01	C-IV	−	−	+	+	−	−	−	+	+	−	−	+	−	+	+	−	−	−	−	+	−

na, Not applicable; IND, indole synthesis; URE, urea; GLU, D-glucose; MAN, D-mannitol; LAC, D-lactose; SAC, D-sucrose; MAL, D-maltose; SAL, salicin; XYL, D-xylose; ARA, L-arabinose; GEL, gelatine; ESC, aesculin hydrolysis; GLY, glycerol; CEL, D-cellobiose; MNE, D-mannose; MLZ, D-melezitose; RAF, D-raffinose; SOR, D-sorbitol; RHA, L-rhamnose; TRE, D-trehalose.

### AMR profiles

Four isolates from cryptic groups C-III and C-IV were tested for resistance to 10 antimicrobials associated with induction of CDI (Table 4). The results demonstrate reduced susceptibility or resistance to the tested cephalosporin antibiotics (ceftriaxone, ceftazidime, cefotaxime, cefepime). The tested isolates also show resistance to the fluoroquinolones ciprofloxacin and levofloxacin (Table 4). A screen of genes from the CARD database identified only a single gene (3003835 *cdeA*) that was observed in cryptic species genomes (BSR values >0.75). A screen for mutations in the *gyrA* and *gyrB* genes associated with fluoroquinolone resistance (mutations listed in the CARD database) indicated the presence of three *gyrB* mutations for the cryptic group isolates (Table S7). While the presence of the *cdeA* gene and these *gyrB* mutations may play a role in resistance to fluoroquinolones. the genomic features conferring resistance to antimicrobial drug families are complex and not well understood, and resistance to ciprofloxacin seems to be common among *C. difficile* with various genomic features [69]. Therefore, the observed resistance to the tested antimicrobials may be due to intrinsic resistance or as yet undescribed acquired genomic features.

**Table 4.** AMR profiles (ETESTs) for Flagstaff isolates. Units are in microgram/milliliter (μg/ml)

Antimicrobial	Antimicrobial class	ES-W-0016-02 (C-III)	ES-S-0010-02 (C-III)	ES-S-0173-01 (C-III)	ES-S-0054-01 (C-IV)	Replicate	R breakpoint	S breakpoint	Reference
Ceftriaxone (CRO)	Cephalosporin	32	48	48	12	Rep1	≥64	≤16	CLSI 2020
		32	48	48	12	Rep2			
Ceftazidime (CAZ)	Cephalosporin	>256	>256	24	32	Rep1	≥64	≤16	CLSI 2020
		>256	>256	24	32	Rep2			
Cefotaxime (CXT)	Cephalosporin	>32	>32	>32	>32	Rep1	≥64	≤16	CLSI 2020
		>32	>32	>32	>32	Rep2			
Cefepime (FEP)	Cephalosporin	>256	>256	>256	>256	Rep1	≥16	≤2	CLSI 2020*
		>256	>256	>256	>256	Rep2			
Clindamycin (CLI)	Macrolide	8	12	2	16	Rep1	≥8	≤2	CLSI 2020
		8	12	2	16	Rep2			
Erythromycin (ERY)	Macrolide	1	1	1	0.25	Rep1	≥8	≤2	EUCAST 2014
		1	1	1	0.25	Rep2			
Imipenem (IMP)	Carbapenem	8	6	6	4	Rep1	≥16	≤4	CLSI 2014
		8	6	6	4	Rep2			
Ciprofloxacin (CIP)	Fluoroquinolone	16	>32	>32	>32	Rep1	≥8	≤2	CLSI 2020†
		16	>32	>32	>32	Rep2			
Moxifloxacin (MXF)	Fluoroquinolone	1.5	1.5	1.5	2	Rep1	≥8	≤2	CLSI 2020
		1.5	1.5	1.5	2	Rep2			
Levofloxacin (LVX)	Fluoroquinolone	8	8	8	16	Rep1	≥2	≤0.5	CLSI 2020*
		8	8	8	16	Rep2			
									
**Susceptible**									
**Intermediate**									
**Resistant**									

*Breakpoint for *Enterobacteriaceae* used.

†Breakpoint for moxifloxacin used.

EUCAST, European Committee on Antimicrobial Susceptibility Testing.

### 16S rRNA and *rpoB* analysis of *Clostridioides*

To determine the ability of single marker genes to resolve *Clostridioides* groups, regions associated with the 16S rRNA gene (Fig. S4) and *rpoB* (Fig. S5) were extracted from genome assemblies in the reference genome set (*n*=101). The 16S rRNA gene provided some resolution between *Clostridioides* clades (Fig. S4a), although the clustering was not fully representative of the core-genome phylogeny. When the 16S rRNA V4 region was extracted from genomes in the reference set, few SNP differences were identified between genomes (Fig. S4b) and most *C. difficile* and cryptic species genomes were indistinguishable. It should be noted that the quality of 16S rRNA gene sequences in some genome assemblies may be poor, which could impact the performance of this locus at differentiating species. The full-length *rpoB* gene, which is 3717 bp in length, provided much better separation between groups than the 16S rRNA gene (Fig. S5a). Phylomark identified several 300 bp candidate regions of the *rpoB* gene that were amenable to Illumina sequencing and provided consistent clade separation (Fig. S5b); the region used to infer the phylogeny in Fig. S5(b) was associated with positions 750 to 1050 in the *rpoB* gene (accession no. CD630_00660).

### *In silico* PCR

To assess the potential performance of PCR assays for detecting *C. difficile* and toxin genes, primers were screened against genome assemblies *in silico* (Table S8). PCR primers for the previously characterized marker unique to *C. difficile* were predicted to amplify across all *C. difficile* as well as all cryptic clades, and amplification for *C. mangenotii* was not predicted. The probe for this assay was not predicted to bind for C-II genomes and one C-V genome (Table S8). This assay detects not only *C. difficile *, but also closely related cryptic lineages. Assays published previously to detect *tcdA*, *tcdB*, *cdtA* and *cdtB* returned variable results. An assay designed to amplify *tcdA* in *C. difficile* [65] was not predicted to amplify *tcdA* sequences for some *C. difficile* and cryptic clade genomes for which ls-bsr results indicate the presence of *tcdA* homologues. A second assay targeting *tcdA* [66] was predicted to amplify *tcdA* sequences in all *C. difficile* genomes identified as containing *tcdA* genes plus two *C. difficile* genomes that ls-bsr did not identify as *tcdA*-positive (these two genomes contain truncated *tcdA* sequences); this assay was predicted to amplify *tcdA* homologues in C-II genomes but not *tcdA* homologues in C-IV genomes. Several PCR assays targeting *tcdB* were tested *in silico*. ls-bsr results indicated 34 reference set genomes contained *tcdB* sequences (17 *C *. *difficile* genomes and 17 cryptic clade genomes). *In silico* amplification prediction indicated that one assay designed to amplify *tcdB* in *C. difficile* [65] may fail to detect some *tcdB* homologues in cryptic species. Two other assays targeting *tcdB* ([66], this study) were predicted to provide more broad amplification of *tcdB* sequences in cryptic clades. Predicted amplification of these two primer sets agree with ls-bsr results except amplification with the primer set generated as part of this study is not predicted for one genome (ERR2215981_ST369, clade C-III) for which ls-bsr results indicate the presence of a *tcdB* homologue. Also, this primer set is predicted to amplify a partial *tcdB* gene sequence present in a genome for which ls-bsr results indicate the absence of the gene. It should be noted that predicted *in silico* PCR amplification and *in vitro* amplification results may differ in cases where a few nucleotide mismatches are present.

## Discussion

*C. difficile* is an important human pathogen associated with potentially serious disease manifestation. Most *C. difficile* studies have focused on the presence of the pathogen in the hospital, especially due to concerns of hospital-acquired infection [70] and antibiotic-associated CDI [71]. However, recent work has identified *C. difficile* spores widely distributed in the environment [4], suggesting a largely understudied reservoir of *C. difficile *. Because of the oxygenated environments where spores have been found and the strictly anaerobic nature of the pathogen [72], most believe that *C. difficile* spores represent a dormant state until a new host is found [73]. Additional surveillance for *C. difficile* in anaerobic environments (e.g. wetlands) may uncover an unknown ecological function besides mammalian infection, although the pathogen is likely adapted to animal hosts.

In this study, we surveyed soil and water in Flagstaff (AZ, USA), to detect *C. difficile* spores; we combined our results with a study focused on the environmental detection of *C. difficile* in Slovenia [4]. Following enrichment, a qPCR (quantitative PCR) assay that targets a chromosomal marker thought to be specific to *C. difficile* was employed for Flagstaff samples; the chromosomal marker was determined to be specific to *C. difficile* based on the analysis of a smaller number of genomes before the identification of the cryptic species [30]. Slovenian strains were isolated without enrichment using chromID *C. difficile* selective agar (bioMérieux). Isolates were identified by typical colony morphology, followed by MALDI-TOF identification and further molecular confirmation with 16S rRNA gene sequencing as described previously [4]. Though different isolation and identification methods were used at each location, whole-genome sequencing followed by an analysis based on ANI demonstrated that some genomes from each location were highly divergent from known *C. difficile* genomes. By including all *Clostridioides* genomes from GenBank, as well divergent genomes collected from environmental sites in Flagstaff and Slovenia, the identification of five divergent clades were identified that were distinct from *C. difficile *.

Pairwise ANI values confirmed that three clades (C-I–C-III) represent potentially novel species, based on an ANI threshold of 95 %; this result was recently published [7], although it doesn’t include the full complement of genomes analysed here. In this study, we publish genomes from an additional clade (C-IV), representing a close, extant near-neighbour species of *C. difficile *. We also demonstrate the presence of an additional novel clade (C-V) that shows pairwise ANI values less than 95 % to the next closest genome, a threshold used previously to delineate bacterial species [39]. Phenotypic information was obtained for isolates from two near-neighbour genomospecies, including information on enzymatic activity and AMR. Observed differences in phenotype, in addition to genomic ANI differences, suggests that these clades in fact represent novel, cryptic species that expand the known genomically characterized diversity of *Clostridioides *.

In clade C-III, substantial genomic differences were observed between genomes collected from the USA and Slovenia (differentiated by thousands of core-genome SNPs; see Fig. 5). These isolates were identified by a relatively limited sampling effort, suggesting that enormous, untapped diversity of cryptic *Clostridioides* likely exists in soil and water worldwide. In this study, we identified genomic signatures that are unique to four of the five clades (C-I, C-III, C-IV, C-V), suggesting that targeted assays could be designed to probe for isolates in these clades. However, there may be other *Clostridioides* isolates in the environment that also contain these signatures, which could further expand the diversity of the genus.

An *in silico* screen of toxin genes demonstrated that there were homologues of *tcdB*, *tcdA*, *cdtA* and *cdtB* in many near-neighbour genomes (>0.5 BSR value, see Methods). For clade C-III genomes sequenced in this study, many genomes were positive for binary toxin homologues (Fig. 2). Binary toxins in *C. difficile* have been associated with increased disease severity [10], and while the role of binary toxins in CDI is still under investigation, binary toxins may impact disease by suppressing the host immune response [74] or increasing adhesion to host cells [75]. The role of the binary toxin homologues in environmental C-III genomes is unknown, but if divergence estimates of this lineage compared to *C. difficile* are accurate, these genes are potentially millions of years old [7] and have an unknown ecological function. In a C-IV genome, the binary toxin homologues were located on a plasmid, although no homologue to *cdtR* was identified. Although intact binary toxin genes have previously been identified on a phage [76], this is the first example of binary toxin genes being located on a plasmid. A screen of all reads from this study demonstrated that the phiSemix9P1 phage, which contains a binary toxin gene locus, was present in multiple (*n*=8) C-III clade genomes from Slovenia, suggesting that the presence of binary toxin genes on mobile elements is much more widespread than previously thought.

Homologues to *tcdB* were identified in multiple cryptic clades, although they demonstrated substantial amino acid differences to *tcdB* in *C. difficile* (Fig. 2). Some clades demonstrated a broad conservation of these genes, whereas other clades demonstrated within-clade diversity, suggesting horizontal acquisition rather than vertical inheritance and divergence. Substantial variability in *tcdB* and horizontal transfer combined with module rearrangement was also described for previously recognized (clade 1–5) *C *. *difficile* strains [15]. Previously, it was suggested that differences in *tcdB* from cryptic species could result in PCR-based assay failures due to nucleotide mismatches [7]. An *in silico* screen indicated that published assays potentially amplify some cryptic *tcdB* and *tcdA* variants (Table S8); however, the diversity of these toxin genes must be considered when attempting to detect clinical infections with these cryptic species if PCR-based assays are used for pathogen detection. The *in silico* PCR requires close primer/probe matches, and an amplicon could perhaps still be generated if single nucleotide mismatches were present. For example, the ‘unique’ *C. difficile* marker used in this study may not be expected to identify some cryptic species using *in silico* PCR (Table S8) due to probe mismatches, directly conflicting with our *in vitro* observations.

Clade C-III genomes contain a *tcdA* homologue that demonstrates significant amino acid differences to *tcdA* in *C. difficile* (Fig. 2). Although *tcdA+/tcdB*− C-III isolates were all from soil, one C-II *tcdA*+/*tcdB−* isolate was associated with antibiotic-induced disease [16]. Future work, potentially using animal models of CDI, will determine the disease potential of these strains.

Previously, the 16S rRNA gene has been used as a method for speciation between closely related species within a genus [77]. In this study, we extracted 16S rRNA genes from all *Clostridioides* genomes and inferred a phylogeny. However, the phylogeny failed to distinguish between *Clostridioides* genomes (Fig. S4a), suggesting that a different marker is required. When just the V4 region of the 16S rRNA gene was extracted, almost no resolution was identified (Fig. S4b). In an environmental survey based on sequence data from this region, V4 amplicons would erroneously identify the presence of *C. difficile* if cryptic species were present instead. The *rpoB* gene has been applied to identify bacterial species [78], and we determined that the *rpoB* gene did a much better job of species differentiation (Fig. S5a), although the longer gene (>3 kb) would be more difficult to sequence and analyse using either Sanger or Illumina sequencing. We did identify a region of the *rpoB* gene that is of a reasonable size for amplicon sequencing on the Illumina platform and generally groups the cryptic species into the correct clades, although the deeper branching order is not consistent with the core-genome phylogeny. Primers could target this region for deep sequencing of environmental samples in the search for previously described cryptic species or potentially new and undiscovered clades.

The addition of these genomes provides a background for designing more specific and sensitive *C. difficile* diagnostics. Previously, we demonstrated how the inclusion of near-neighbour genomes results in an erosion of the diagnostic signature space [79, 80], but is critical for environmental surveillance. Although the ecology of isolates from these cryptic species is largely unknown, an investigation into unique genes may provide insight into the function of these isolates. As the cultures were grown in anaerobic conditions, but were collected from surface soils and water, an assumption is that these spores were dormant in the environment. Additional work looking at oxygen tolerance in isolates from these lineages may identify differences to *C. difficile *, representing a different evolutionary history and a unique ecological role.

The results of this work significantly expand the genomic landscape of *Clostridioides *. Although some of the genomes from cryptic species were previously published, this study adds significant genomic diversity to the genus. Divergent strains are frequently associated with atypical variants of all four toxin genes (*tcdB*, *tcdA*, *cdtA*, *cdtB*), which are inserted in different chromosomal regions than the PaLoc or CdtLoc or are associated with mobile genetic elements (e.g. plasmids). The isolates collected, sequenced and analysed in this study were from just two locations and of limited geographical and temporal diversity. A targeted survey of the environment from multiple states in the USA and multiple countries in Europe and Asia is expected to expand our understanding of these cryptic species, perhaps adding additional context into the evolution and diversification of a globally distributed human pathogen.

## Supplementary Data

Supplementary material 1

Supplementary material 2
