# Supplementary material for: Identification of novel, cryptic Clostridioides species isolates from environmental samples collected from diverse geographical locations
Source: Microb Genom. 2022 Feb 15;8(2):000742. doi: 10.1099/mgen.0.000742 (PMC8942030; doi:10.1099/mgen.0.000742)
Supplement: Supplementary material 1 [file mgen-8-0742-s001.pdf]

Supplementary figures and table information for "Identification of novel, cryptic *Clostridioides* species isolates from environmental samples collected from diverse geographical locations"

__Supplemental Figures__

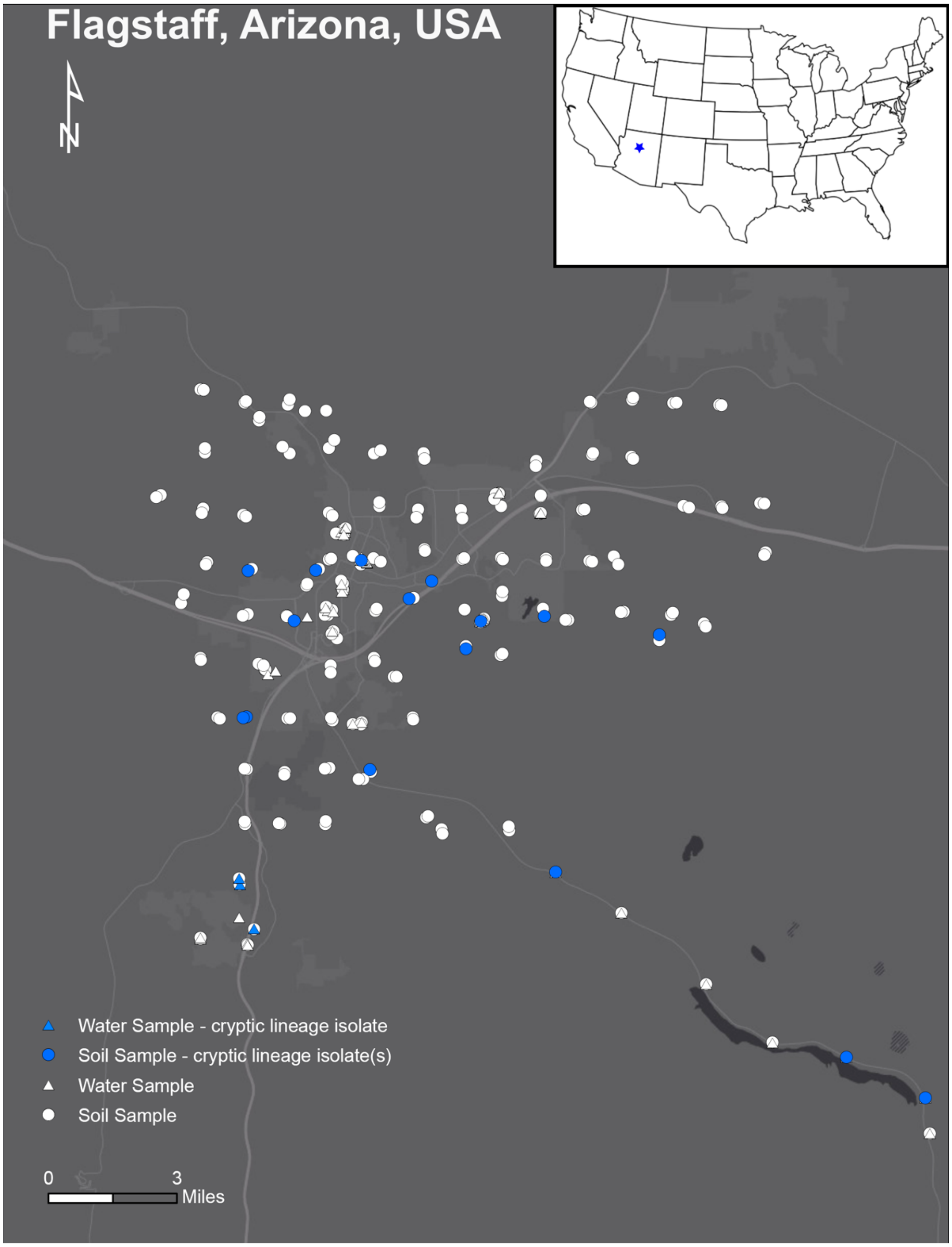

Figure S1. Map of sampling sites for detection of *C. difficile* and cryptic lineage isolates in environmental samples in Flagstaff, Arizona, USA. Triangles denote water samples; circles denote soil samples. Samples from which cryptic lineages were isolated are highlighted in blue.

Genome	Plasmids								Phage						Clade
	pESS10701b	pESS10701c	pESS10701d	pESS17301a	pESS17301c	pESS17301d	pESS17301e	pESW1602	LN681534.1	KX905163.1	ES-S-0173-01	ES-S-0107-01	pCD5401_3	pCD1602_4	
ES-S-0173-01	10	3	1	100	100	100	100	0	3	3	100	0	4	1	C-III
ES-S-0001-02	6	0	13	0	16	12	6	0	65	46	1	0	8	1	C-III
ES-S-0001-03	22	0	19	0	51	73	79	2	68	57	4	0	16	3	C-III
ES-S-0005-03	11	0	2	0	100	73	5	98	22	4	100	0	6	2	C-III
ES-S-0006-03	8	0	3	0	16	25	12	1	68	54	19	9	6	45	C-III
ES-S-0123-01	9	0	30	0	4	7	1	0	69	2	0	0	10	2	C-III
ES-W-0016-02	3	0	1	0	2	0	0	100	24	5	3	2	4	100	C-III
ES-W-0017-02	10	0	16	0	16	27	42	2	3	63	69	14	32	37	C-III
ES-W-0018-02	10	0	16	0	16	27	42	2	3	63	69	14	31	37	C-III
ZZV13-5731	6	0	14	0	1	30	14	2	74	100	2	2	8	3	C-III
ZZV14-5902	7	21	31	3	2	31	20	2	2	100	28	17	4	28	C-III
ZZV14-6009	4	0	4	0	2	3	44	2	1	100	34	16	10	30	C-III
ZZV14-6044	6	55	29	3	2	32	49	2	74	100	3	2	5	4	C-III
ZZV14-6045	9	1	31	0	2	31	13	74	73	100	27	18	5	28	C-III
ZZV14-6048	6	20	16	3	2	30	45	2	2	100	38	15	4	35	C-III
ZZV14-6104	4	0	6	0	2	30	5	2	74	100	3	2	4	3	C-III
ZZV14-6105	4	0	11	0	5	0	0	2	73	72	2	1	3	3	C-III
ZZV14-6150	2	0	4	0	2	3	42	2	59	100	13	22	4	14	C-III
ZZV14-6154	3	0	5	0	1	30	4	2	75	1	2	1	9	4	C-III
ES-S-0107-01	100	100	100	5	3	19	2	1	1	4	2	100	15	4	C-IV
ES-S-0108-01	100	100	100	5	3	20	2	1	1	4	2	100	14	4	C-IV
ES-S-0054-01	10	3	0	1	1	0	2	1	1	0	2	1	100	1	C-IV
ES-S-0056-01	12	3	0	1	1	2	2	1	3	2	2	2	87	1	C-IV
ES-S-0077-01	12	3	3	1	1	2	2	1	3	2	2	2	87	1	C-IV

Figure S2. Breadth of coverage values obtained from mapping sequencing reads for all genomes sequenced as part of this study to extrachromosomal elements.

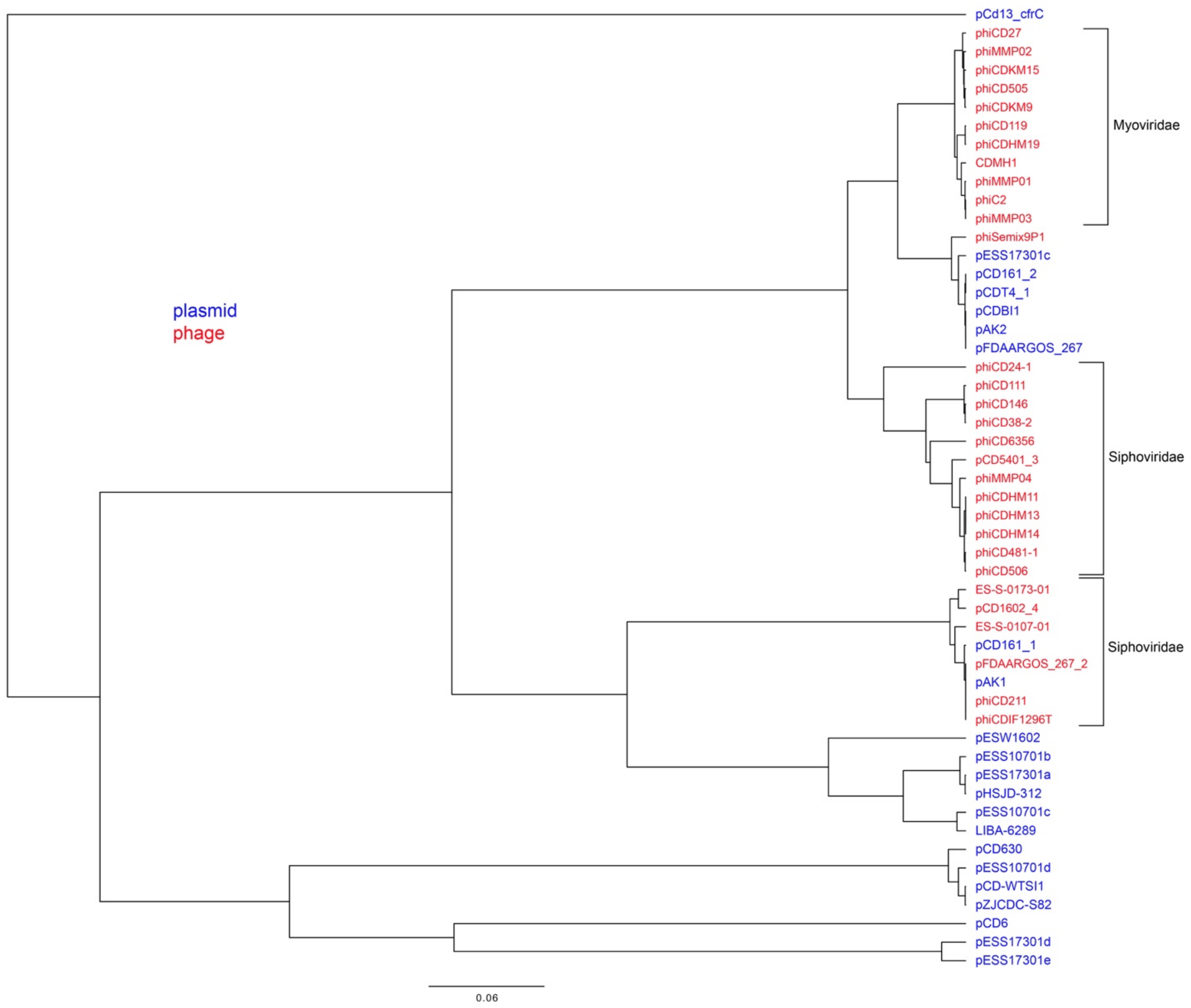

Figure S3. Dendrogram displaying relationships of extrachromosomal elements based on pairwise MASH distances.

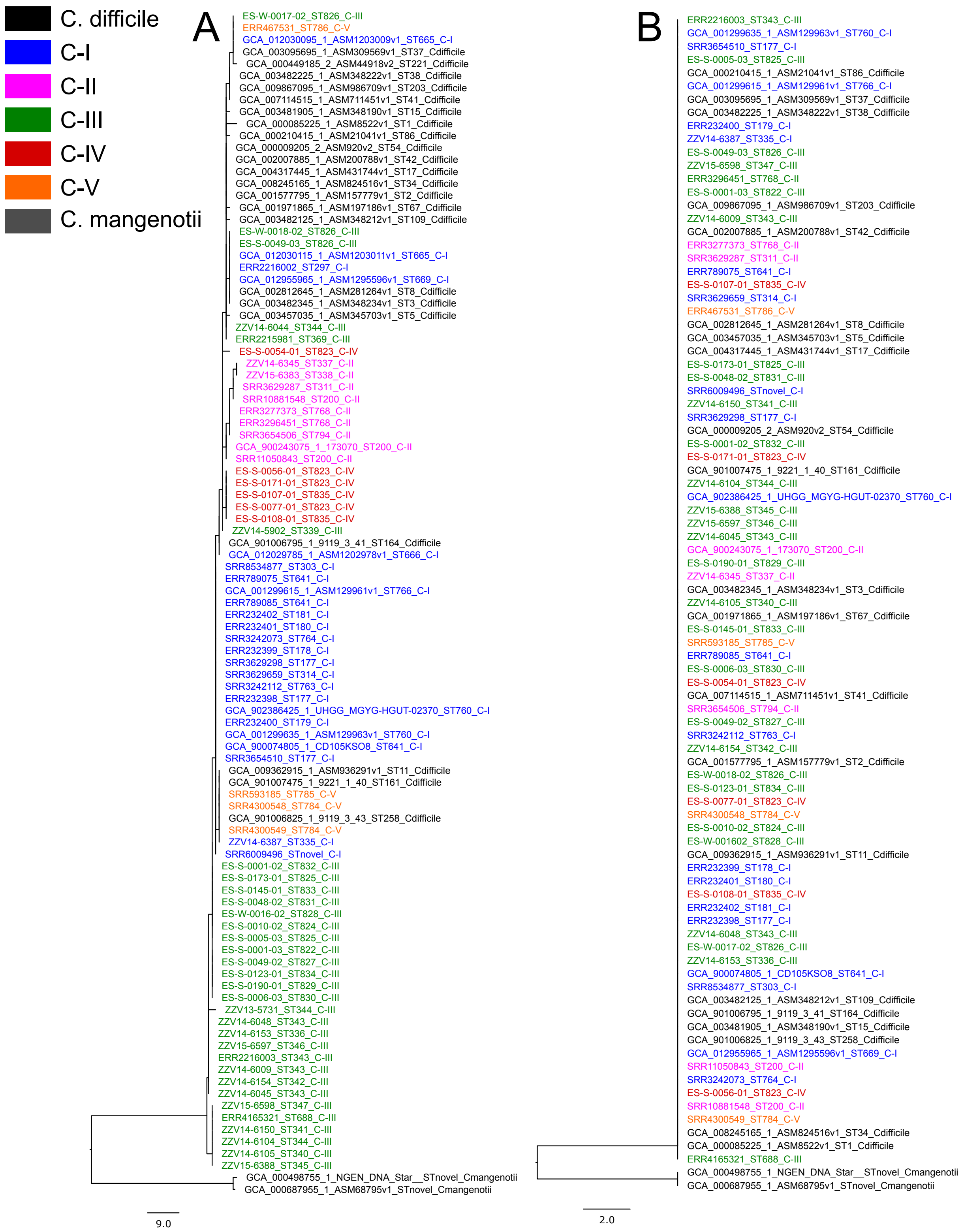

Figure S4. A) Phylogeny of near-full-length 16S rRNA sequences. B) Phylogeny of ~300bp V4 region of the 16S rRNA gene.

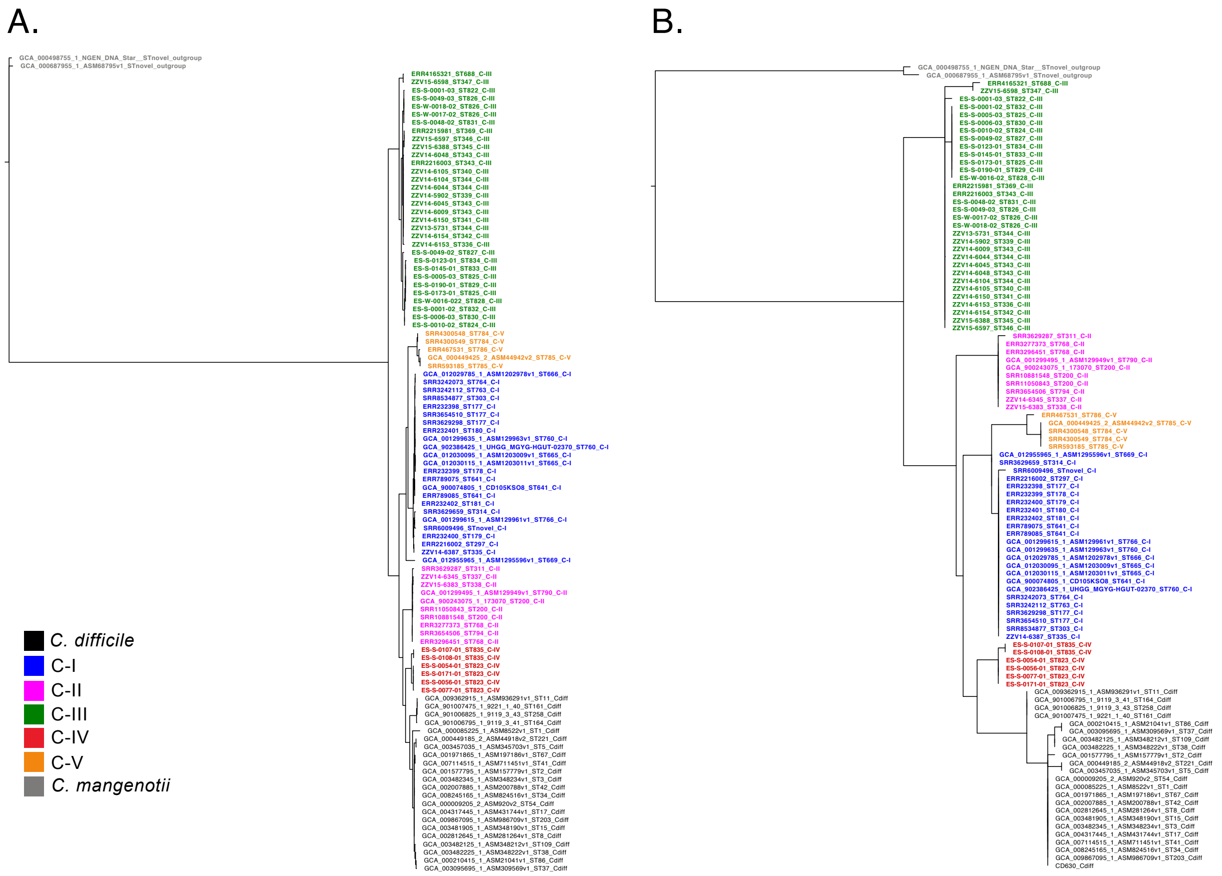

Figure S5. A) Phylogeny of near-full-length *rpoB* gene sequences extracted from genome assemblies. B) Phylogeny of 300bp region of the *rpoB* gene identified by Phylomark as a potential marker suitable for delineating cryptic species.

Supplemental Tables

Supplemental tables are available as an additional file (spreadsheet with multiple tabs).

Table S1. Accession numbers and genome assembly information for newly sequenced genomes.
Table S2. Accession numbers and genome assembly information for publicly available genomes included in this study.
Table S3. Primers for PCR assays investigated in this study.
Table S4. Average nucleotide identity values generated with PYANI.
Table S5. Lineage-specific sequences identified for cryptic clades using LS-BSR.
Table S6. Annotation information for lineage-specific coding regions and lost coding coding regions associated with clade C-III.
Table S7. Mutations in *gyrB* associated with fluoroquinolone resistance identified for cryptic group isolates.
Table S8. Results of *in silico* screen of primers for PCR assays investigated in this study.
